# Supplementary material for: Phosphatase activity tunes two-component system sensor detection threshold
Source: Nat Commun. 2018 Apr 12;9:1433. doi: 10.1038/s41467-018-03929-y (PMC5897336; doi:10.1038/s41467-018-03929-y)
Supplement: Supplementary file 1 — Supplementary Information [file 41467_2018_3929_MOESM1_ESM.pdf]

Supplemental text and figures

Tuning bacterial two-component system input detection thresholds

Landry et al.

Supplementary Note 1. Model of the effect of phosphatase activity on detection threshold.

1. Derivation of [RR~P]

The Batchelor-Goulian TCS model[1] depicts reactions for SK autophosphorylation/SK~P autodephosphorylation, SK~P/RR binding/unbinding, transfer of the phosphoryl group from SK~P to RR, SK/RR~P binding/unbinding, and dephosphorylation of RR~P by SK. The reactions, rate constants, and corresponding ordinary differential equations are depicted below:

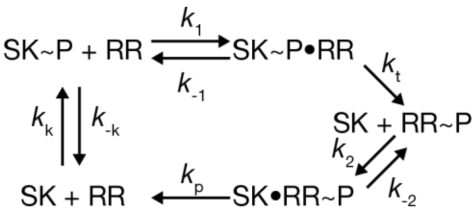

$$\frac{d[SK{\sim}P\cdot RR]}{dt} = k_1[SK{\sim}P][RR] - (k_{-1} + k_t)[SK{\sim}P\cdot RR] \tag{1}$$

$$\frac{d[SK\cdot RR{\sim}P]}{dt} = -(k_p + k_{-2})[SK\cdot RR{\sim}P] + k_2[SK][RR{\sim}P] \tag{2}$$

$$\frac{d[SK{\sim}P]}{dt} = k_k[SK] - k_{-k}[SK{\sim}P] + k_{-1}[SK{\sim}P\cdot RR] - k_1[SK{\sim}P][RR] \tag{3}$$

$$\frac{d[SK]}{dt} = k_{-k}[SK{\sim}P] - k_k[SK] + \left(k_p + k_{-2}\right)[SK\cdot RR{\sim}P] + k_t[SK{\sim}P\cdot RR] - k_2[SK][RR{\sim}P] \tag{4}$$

$$\frac{d[RR]}{dt} = k_{-1}[SK{\sim}P\cdot RR] - k_1[SK{\sim}P][RR] + k_p[SK\cdot RR{\sim}P] \tag{5}$$

$$\frac{d[RR{\sim}P]}{dt} = k_t[SK{\sim}P\cdot RR] + k_{-2}[SK\cdot RR{\sim}P] - k_2[SK][RR{\sim}P] \tag{6}$$

Following the authors, we assume that total SK (i.e. $[SK]_T$) and RR (i.e. $[RR]_T$) are constant and write:

$$[SK]_T = [SK{\sim}P\cdot RR] + [SK\cdot RR{\sim}P] + [SK{\sim}P] + [SK] \tag{7}$$

$$[RR]_T = [SK{\sim}P\cdot RR] + [SK\cdot RR{\sim}P] + [RR{\sim}P] + [RR] \tag{8}$$

Then, at steady state, all differential equations equal zero and:

$$[RR{\sim}P] = \frac{C_p[RR]}{C_t + [RR]} \tag{9}$$

Where concentrations C_t and C_p are the composite rate constants and CE_t and CE_p represent the catalytic efficiencies (k_{cat}/K_m; $sec^{-1}\,M^{-1}$) of the Michaelis reactions of kinase and phosphatase activity[2]:

$$C_t = k_{-k} * \frac{k_{-1} + k_t}{k_1 k_t} = \frac{k_{-k}}{CE_t} \quad (10) \qquad C_p = k_k \frac{k_{-2} + k_p}{k_p k_2} = \frac{k_k}{CE_p} \quad (11)$$

In the limit where $[SK]_T << [RR]_T$, equation (8) can be substituted into equation (9) to give the following quadratic solution for [RR~P][1]:

$$[RR{\sim}P] = \frac{1}{2}\left(C_t + C_p + [RR]_T\right) - \frac{1}{2}\sqrt{\left(C_t + C_p + [RR]_T\right)^2 - 4C_p * [RR]_T} \quad (12)$$

2. Modulation of phosphatase activity

SK phosphatase activity can be described as a Michaelis reaction where the SK reversibly binds RR~P and then irreversibly removes a phosphate. The catalytic efficiency (CE) of such a reaction is defined as k_{cat}/K_m, which is equivalent to CE_p. CE is a useful metric when comparing different enzymatic activities, such as the phosphatase and kinase activity of a SK on its cognate response regulator. A large number of point mutations have been found in SKs that predominantly decrease the phosphatase activity, and therefore CE_p of the enzyme, as compared to CE_t. Since the individual rate constants of a TCS cannot be measured accurately, we modeled the effect of phosphatase-diminishing mutations by scaling the value of CE_p, which is linearly related to the measurable C_p parameter. We parameterized the above model using previously measured constants for the PhoBR TCS[2] :

$$\frac{k_{-k}}{CE_t} = C_t = 0.8\mu M, \qquad \frac{k_k}{CE_p} = C_p = 4\mu M, \qquad RR_T = 10\mu M$$

3. Model of gene regulation

In a canonical TCS, phosphorylation induces RR dimerization and subsequent promoter binding and regulation of transcription. We modelled these processes via a classical Hill function for activatable promoters. In this function, b represent leaky transcription from the promoter in the absence of activator, and $(b + a)$ is the maximal transcription from the promoter. K_m is the equilibrium constant and the promoter is half activated when RR~P equals K_m. The hill coefficient, n, represents the level of cooperativity of RR~P binding.

$$Transcription = b + a * \frac{RR{\sim}P^n}{K_m^n + RR{\sim}P^n} \quad (13)$$

We used the following parameters to simulate a leaky output promoter.

$$a = 0.9, \quad b = 0.1, \quad K_m = 1\,M, \quad n = 2$$

4. TCS simulations

To simulate the TCS transfer function, we assumed that ligand concentration was linearly related to the autokinase rate of the SK (k_k), as previously[3], scaling k_k between 10^{-2} and 10^{1} (**Fig. 1b**). To simulate the effect of phosphatase-diminishing mutations, we varied the CE_p term between 10^{-2} and 10^{2} fold that of WT. Python code for the simulations is included as Supplementary Material.

5. Relationship between $K_{1/2}$ and dynamic range

We also examined the effect changes in phosphatase activity had on the dynamic range of the TCS. We limited the effect of ligand induction on autokinase activity to the previously mentioned scaling range of 10^{-2} to 10^{1} of basal activity. The dynamic range was then calculated as the ratio of the maximal promoter activity to the basal promoter activity. We found there was a region of phosphatase activity where the detection threshold could be changed without effecting the dynamic range (**Supplementary Fig. 1**). However, large decreases in phosphatase activity resulted in basal levels of RR~P that were high enough to activate the promoter, resulting in basal transcription and decreased dynamic range. Conversely, large increases in phosphatase activity caused RR~P levels to remain low even at high induction, causing decreased maximal expression from the TCS and decreased dynamic range.

6. Analysis of the effect of kinase activity on detection threshold

We also examined the effect that changes in kinase activity could have on the detection threshold of a TCS. Here, rather than increasing SK autophosphorylation (k_k) as before (**Fig 1b; Supplementary Fig. 1**), we simulated the effect of inducer by decreasing SK autodephosphorylation (k_{-k}) between 10^{3} and 10^{0}. With this change, we found that kinase activity had similar control of detection threshold to that previously shown with phosphatase activity; however, here decreases in kinase activity resulted in increases in detection threshold and increases in kinase activity resulted in decreases in detection threshold (**Supplementary Fig. 1**). This finding suggests that either phosphatase activity or kinase activity could serve as a tuning knob for detection threshold. In this work, we focus on phosphatase activity for decreasing detection threshold since this is easier to achieve by weakening phosphatase activity than strengthening kinase activity.

Supplementary Note 2. Predictive model of soil nitrate concentrations.

To predict the concentration of nitrate in soil we collected nitrate transfer functions in soil on three separate days (**Supplementary Fig. 12**). We observed significant day to day variability in the high and low values of the cultures, and therefore normalized each day's data to the high and low for that day. Data from all days was fit with a hill function to obtain best fit parameters (**Methods**).

To predict the nitrate concentration in fertilized soil based on GFP measurements we inverted the hill function to obtain the following equation:

$$Nitrate = \; k * \sqrt[n]{\frac{r}{1-r}}, where \; r = \frac{[GFP] - low}{high - low} \tag{12}$$

Fertilizer transfer functions in soil were taken and GFP levels were measured (**Supplementary Fig. 12e**). The parameterized inverted Hill functions of the NarX and NarX(C415R) TCSs were used to predict nitrate concentrations as shown in **Fig. 6b**. Data points with predicted nitrate concentrations outside of the range of the y-axis were not included in this plot. Accurate detection ranges of the two TCSs were defined as the range in which the predicted nitrate values were within 2-fold of the manufacturer supplied nitrate value.

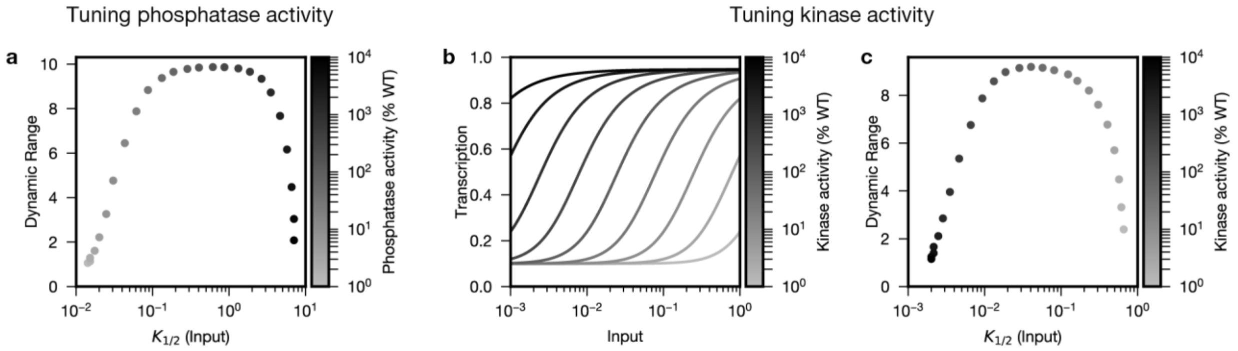

Supplementary Figure 1. Relationship between detection threshold and dynamic range of a TCS.

(**a**) The relationship between the detection threshold and dynamic range of the transfer functions simulated in (**Fig. 1b**). (**b**) Model simulations of the relationship between TCS input concentration and transcriptional output rate (i.e. transfer function) wherein SK kinase activity is varied between 1% and 10,000% of wild-type (**Supplementary Note 1**). (**c**) The relationship between the detection threshold and dynamic range of the transfer functions simulated in Panel B.

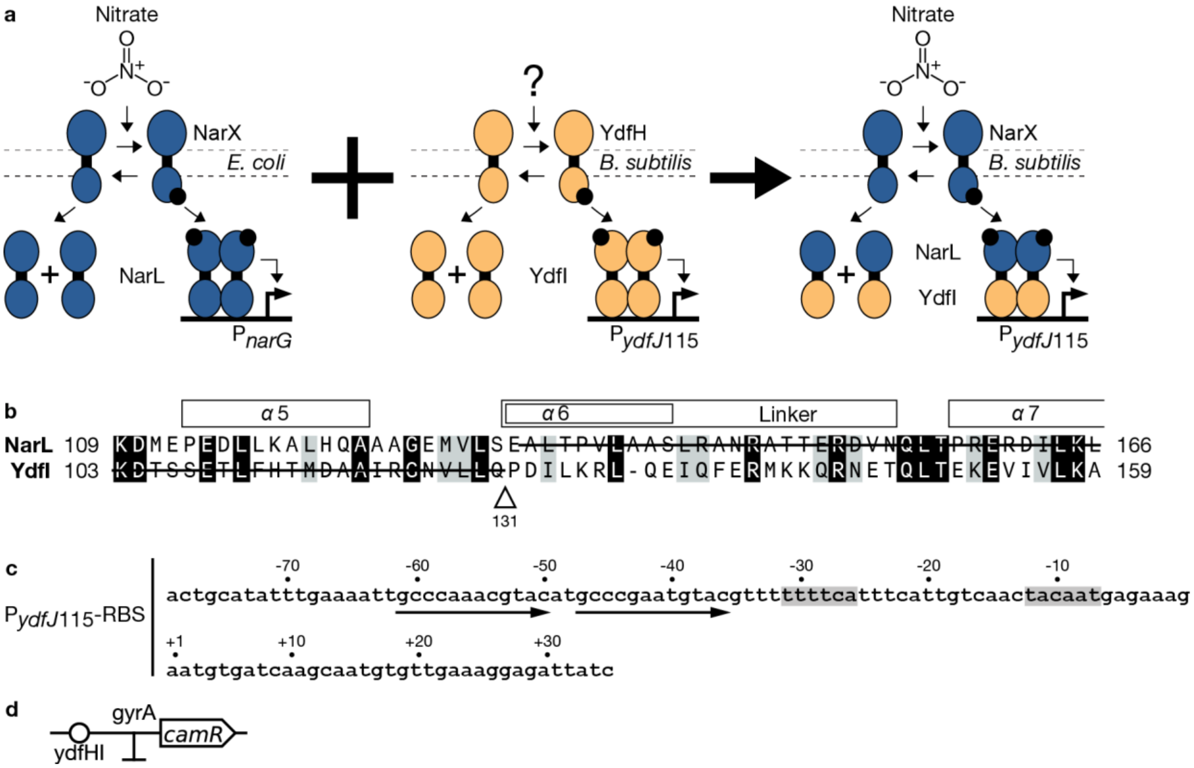

Supplementary Figure 2. Design of the chimeric NarL-YdfI response regulator and its output promoter.

All *B. subtilis* genetic systems engineered in this paper (**Supplementary Table 4**) are integrated into the chromosome. The genetic systems are first constructed as linear double stranded DNA fragments that we call Integration Modules (IMs) prior to integration (**Methods**). We utilize the naming convention "iABxxx" where "i" indicates that the construct is an IM, "AB" indicates the first and last initial of the individual who designed the IM, and "xxx" is a unique numerical identifier. (**a**) We fused the REC domain of NarL to the DBD of YdfI to connect the *E. coli* NarXL TCS to transcription from the *B. subtilis* $P_{ydfJ115}$ promoter. We selected the YdfI DBD due to the high degree of homology of YdfI to NarL and because YdfI regulates a single promoter, P_{ydfJ}, in *B. subtilis*[4], thus reducing the likelihood of unwanted fan-out cross-regulation. P_{ydfJ} is known to be regulated only by YdfI, and thus does not naturally respond to nitrate. The host YdfHI TCS, whose natural ligand is unknown, was knocked out using the IM in Panel D to prevent its regulation of the $P_{ydfJ115}$ promoter. (**b**) An alignment of the linker region of the NarL and YdfI proteins. The residue at which the proteins were joined is shown with a triangle. Identical and similar residues are highlighted with black and gray respectively. (**c**) The NarL-YdfI regulated P_{ydfJ} promoter. YdfI operator sites are shown with an arrow and the -35 and -10 sequences are highlighted with gray.

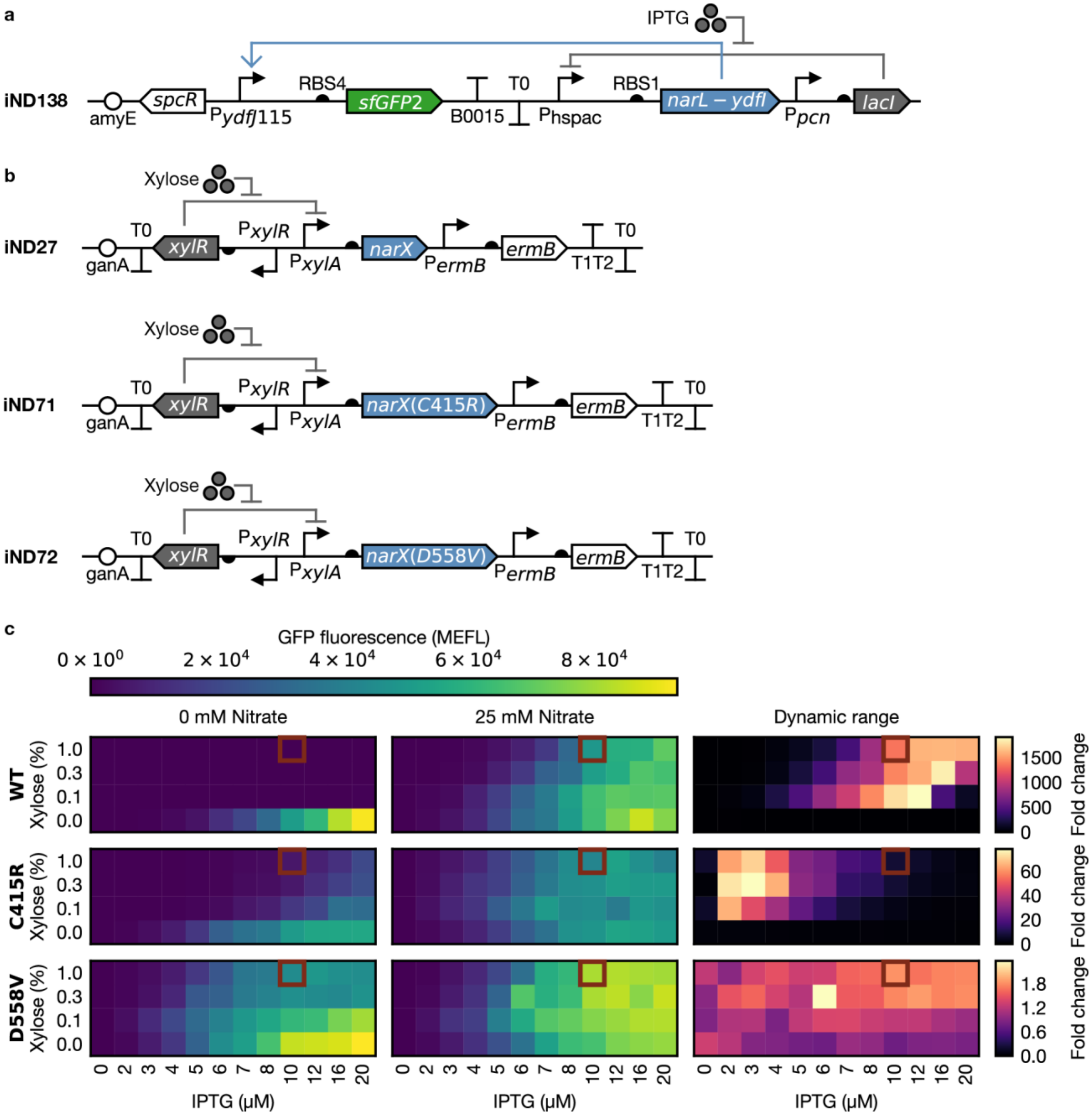

Supplementary Figure 3. Optimization of SK and RR expression levels for *B. subtilis* nitrate sensing TCSs.

(**a**) We constructed iND138 wherein NarL-YdfI (**Supplementary Fig. 2**) is expressed from an IPTG-inducible promoter and sfGFP is expressed from the NarL-YdfI~P activated $P_{ydfJ115}$ output promoter (**Supplementary Fig. 2**). iND138 is integrated into the *amyE* locus. (**b**) NarX, NarX(C415R), and NarX(D558V) are expressed from a xylose-inducible promoter in iND27, iND71, and iND72, respectively. Each of these IMs is integrated into the *ganA* locus of a strain also containing iND138, resulting in a complete TCS. (**c**) We induced the expression of NarL-YdfI and each NarX variant to different extents in two dimensions in the presence and absence of nitrate, measured the resulting sfGFP output, and calculated the dynamic range, or ratio of sfGFP

fluorescence in each condition. To compare detection thresholds of the wild-type and mutant sensors (**Fig. 2**), we selected a single set of induction conditions that resulted in large dynamic range of all three sensors (10µM IPTG, 1% xylose; brown boxes).

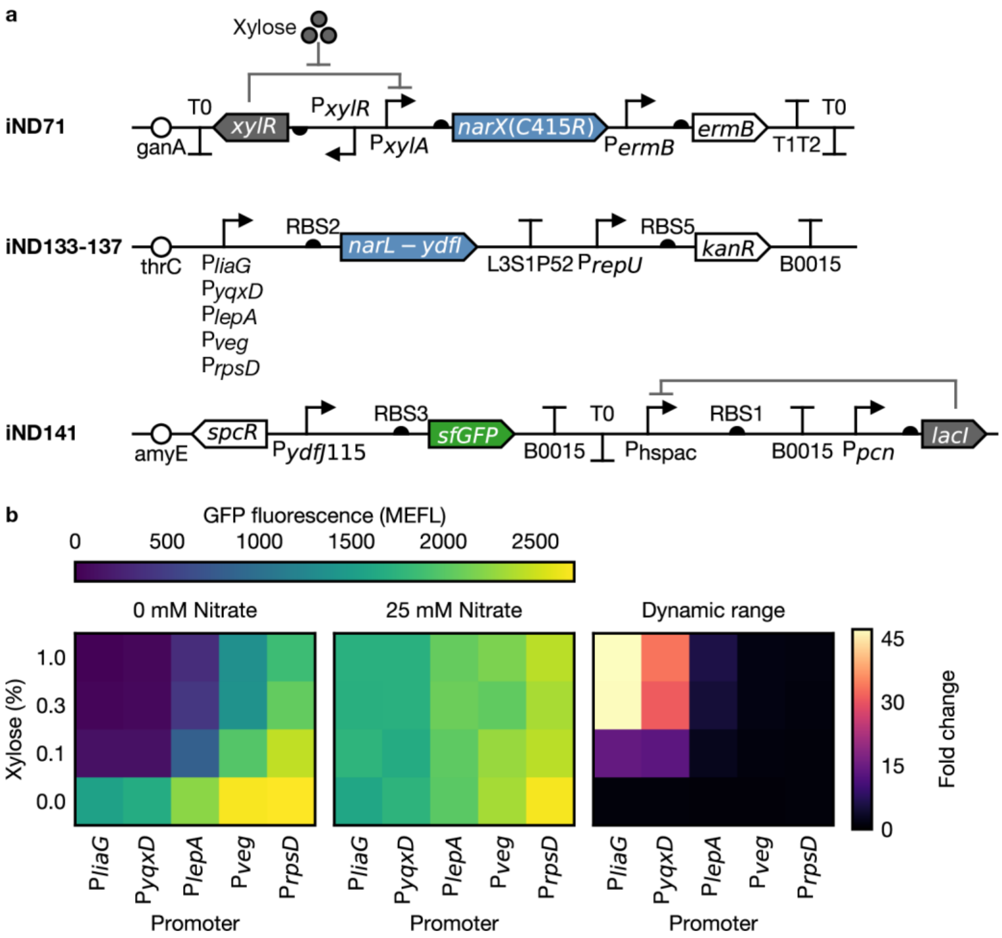

Supplementary Figure 4. Engineering the iso-SK expression strain.

For the iso-SK experiment, we aimed to utilize the IPTG and xylose induction modules (**Supplementary Fig. 3**) to control NarX and NarX(C415R) expression, respectively. However, because we lacked other inducible promoter systems, we first replaced the xylose-inducible NarL-YdfI promoter (**Supplementary Fig. 3**) with a constitutive version. (**a**) We utilized xylose inducible NarX(C415R) and differentially applied nitrate in the media to screen five constitutive *B. subtilis* promoters (P_{liaG}, P_{yqxD}, P_{lepA}, P_{veg}, and P_{rpsD}) for the ability to drive appropriate levels of NarL-YdfI expression. Note that the translation rate of RBS3-sfGFP (**Supplementary Table 2**) is weaker than the translation rate of RBS4-sfGFP2 since this construct was made prior to optimization of *sfgfp* translation, resulting in lower GFP levels when compared to other data in this paper. (**b**) NarX(C415R)/NarL-YdfI performance at different SK and RR expression levels. We selected P_{liaG}, which gave the highest fold change, for the iso-SK experiment.

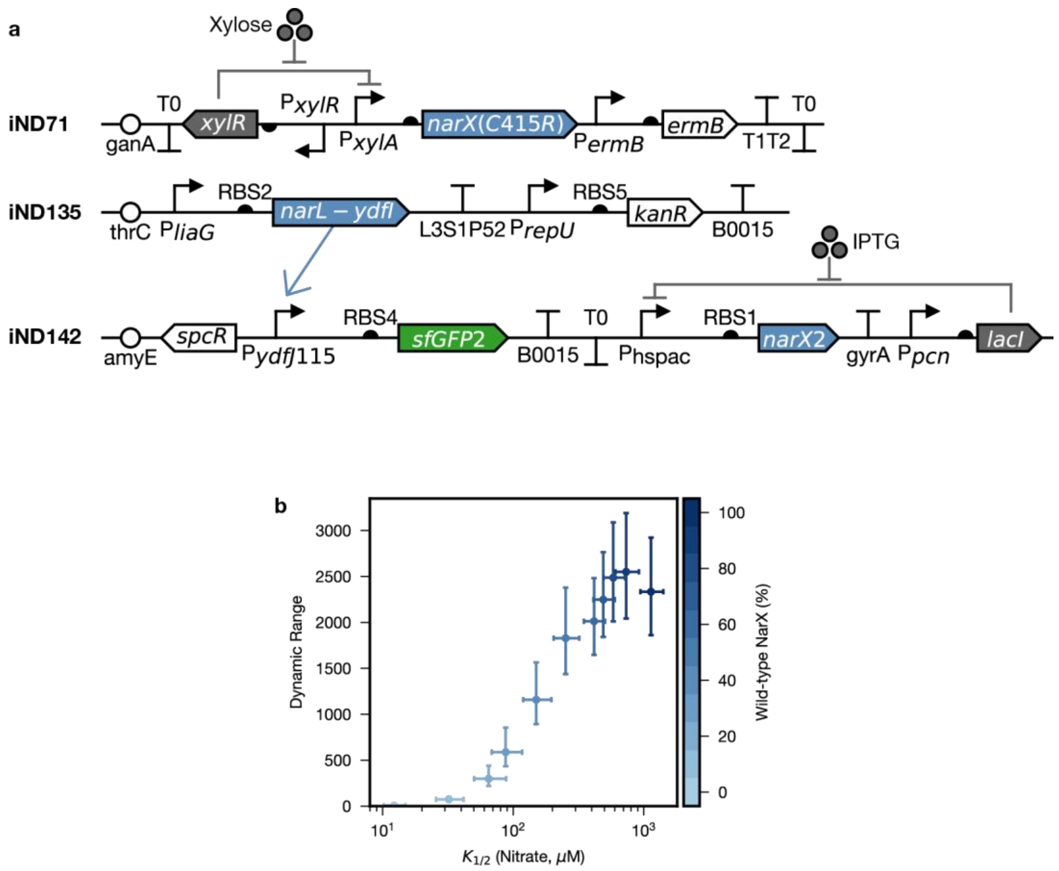

Supplementary Figure 5. Performance of the iso-SK expression strain.

(**a**) The iso-SK strain device schematics. (**b**) The relationship between the detection threshold and dynamic range of the iso-SK strain at different expression levels. Points represent the best fit value of the parameter and error bars the 95% confidence interval of the fit parameter.

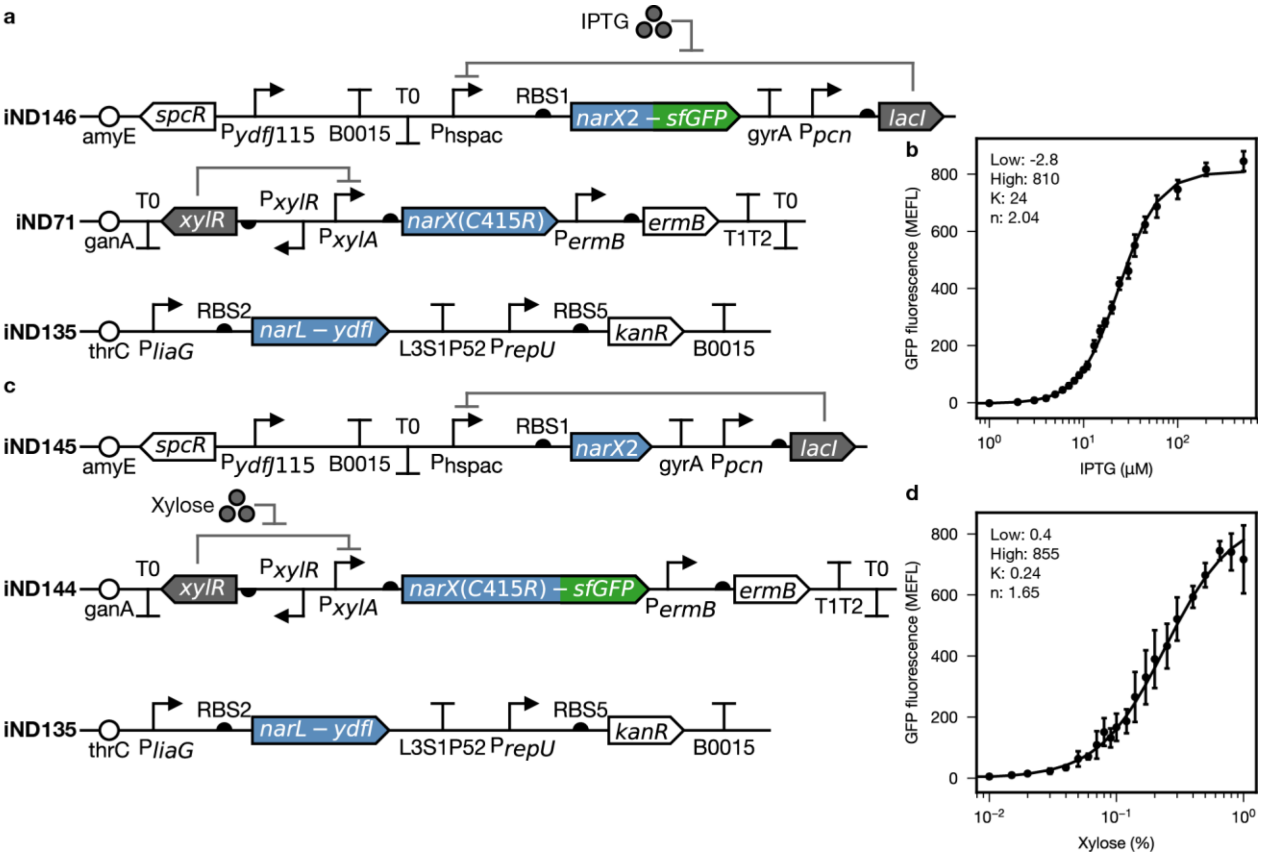

Supplementary Figure 6. Quantification of NarX and NarX(C415R) expression levels for the iso-SK experiment.

(**a, c**) To quantify the relationship between IPTG and NarX expression and xylose and NarX(C415R) expression, we fused sfGFP to the C-terminus of each SK in separate test strains. (**b,d**) We then measured the IPTG and xylose transfer functions (**Methods**). Data points are the mean and error bars are the SEM of experiments on three separate days. Each data set is fit to a transfer function model (**Methods**).

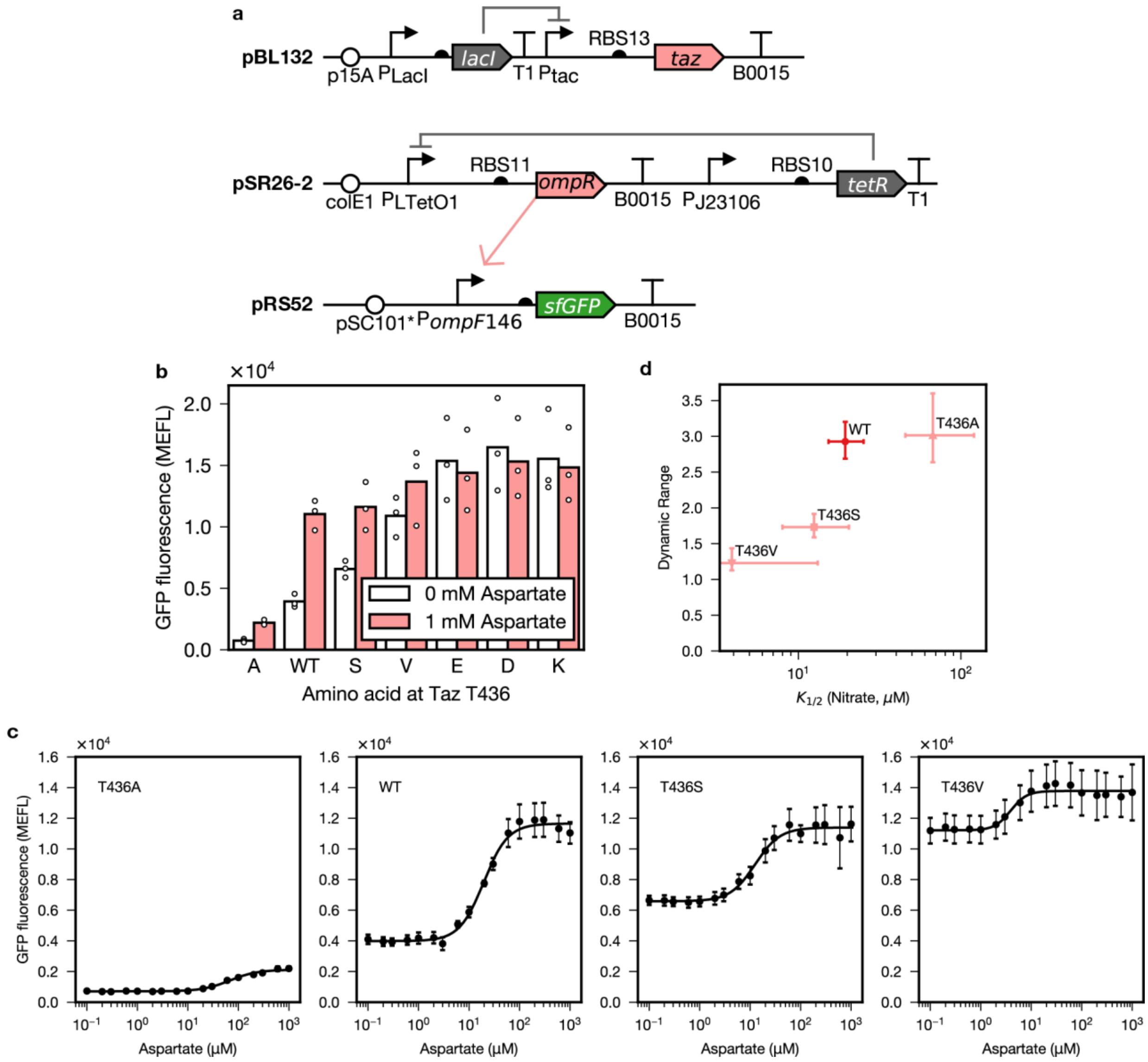

Supplementary Figure 7. Tuning the detection threshold of the aspartate sensing Taz-OmpR system by mutating the phosphatase hot spot.

(**a**) Device schematic of the Taz-OmpR system utilized in this paper. Plasmid names are shown. (**b**) Output of Taz-OmpR with wild-type and previously-characterized mutations at the phosphatase hot spot (T436)[5] in the absence and presence of aspartate. Circles are raw data and bars are means from experiments on three separate days (**Methods**). (**c**) Aspartate transfer functions of the four functional Taz-OmpR systems from panel 'b'. Data points, error bars, and model fitting are as described in **Supplementary Fig. 6**. (**d**) The relationship between the detection threshold and dynamic range of the transfer functions in panel 'c'. Data points and error bars, are as described in **Supplementary Fig. 5**.

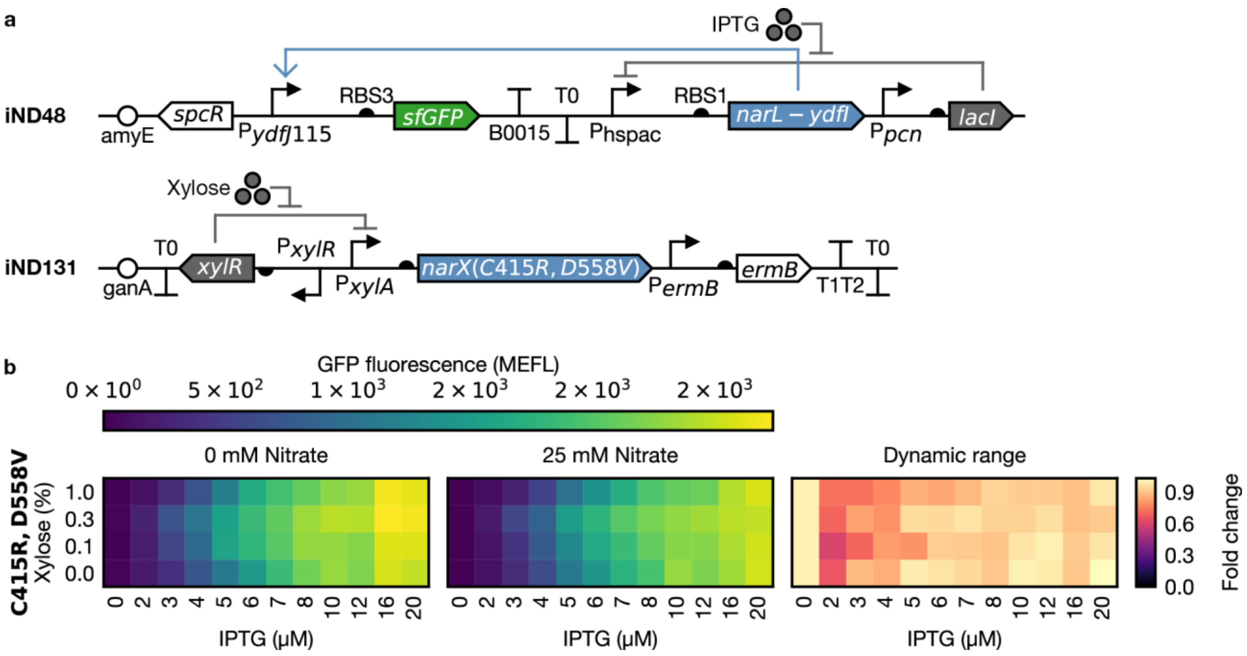

Supplementary Figure 8. The NarX(C415R, D558V) double mutant does signal in response to nitrate.

(**a**) Device schematic of NarX double mutant TCS system. (**b**) TCS output and fold change at different IPTG and xylose concentrations. There is little observable effect of SK induction on RR function with or without nitrate, suggesting that signaling has been abolished due to the combined effects of two phosphatase-reducing mutations. Note that iND48 contains the weakly translating RBS3-sfGFP (**Supplementary Table 2**) as in **Supplementary Fig. 4,** resulting in lower GFP levels when compared to other data in this paper.

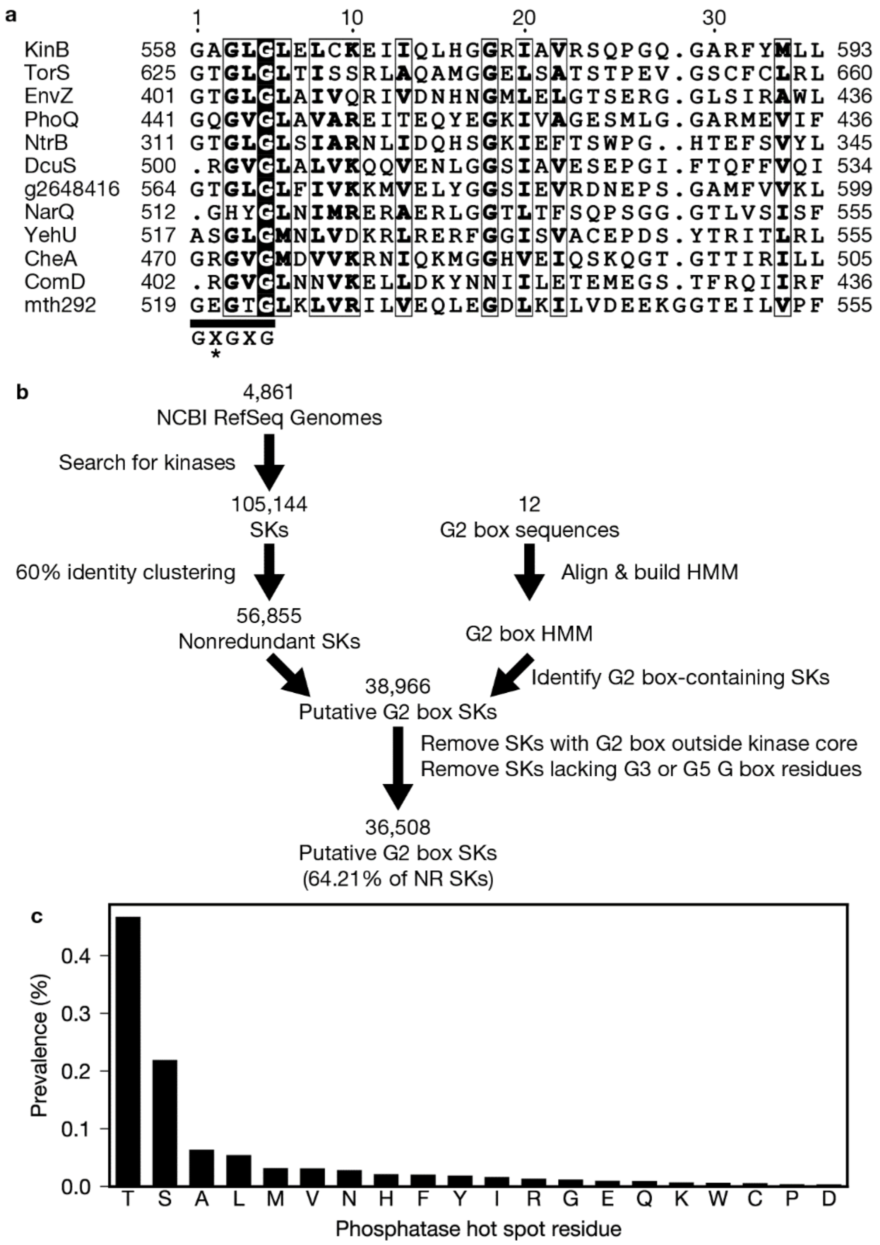

Supplementary Figure 9. The phosphatase hot spot residue is present in 64% of SKs.

(a) Development of the Hidden Markov Model (HMM) (**Fig. 5a**) of the conserved CA domain G2 box region containing the GXGXG motif (**Methods**). An alignment of 12 SKs from diverse sub-families that contain the G2 box (redrawn from Wolanin et al.[6]). The GXGXG motif is underlined and the phosphatase hot spot residue within the motif is indicated (*). The bold white "G" on black background is conserved in all 12 SKs. Boxes indicate positions with greater than 70% conservation of similar residues across all 12 SKs. Bold residues are similar to 70% of other residues in the same column. (**b**) We identified 56,855 non-redundant SKs from genomes in the NCBI RefSeq database (**Methods**). Using the G2 box HMM, we determined that 38,966 of these SKs contain the G2 box region. Then, we eliminated those SKs wherein the G2 box region was outside the kinase core, which is composed of the DhP and CA domains, or that lacked glycines

at either of the final two conserved GXGXG positions. This restriction yielded 36,508 SKs (64% of non-redundant SKs) that contain the phosphatase hot spot residue. (**c**) The distribution of amino acids found at the phosphatase hot spot residue.

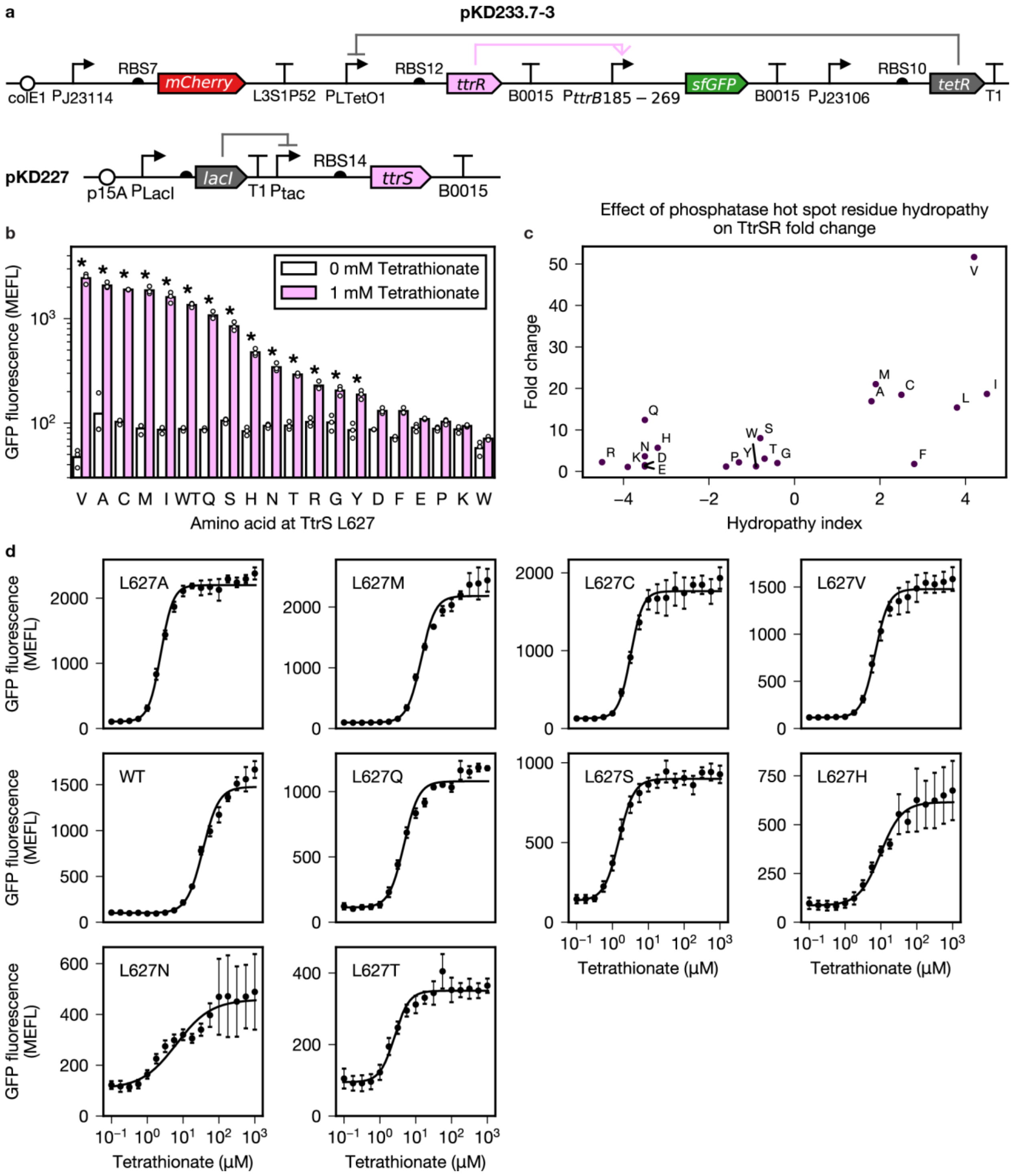

Supplementary Figure 10. Detailed characterization of TtrS phosphatase hot spot mutants.

(**a**) Device schematic of *E. coli* TtrSR system used in this work. The mCherry transcriptional unit has no function in this work. (**b**) Tetrathionate response of TtrSR with all 20 amino acids at the TtrS phosphatase hot spot. We define functional mutants (*) as those with fold activation > 2. Circles and bars are as described in **Supplementary Fig. 7**. (**c**) Relationship between

hydropathy[7] of the TtrS phosphatase hot spot residue and the tetrathionate response of the TCS. (**d**) Tetrathionate transfer functions of the ten L627 mutants with the largest fold activation. Data points, error bars, and model fits are as described in **Supplementary Fig. 6**.

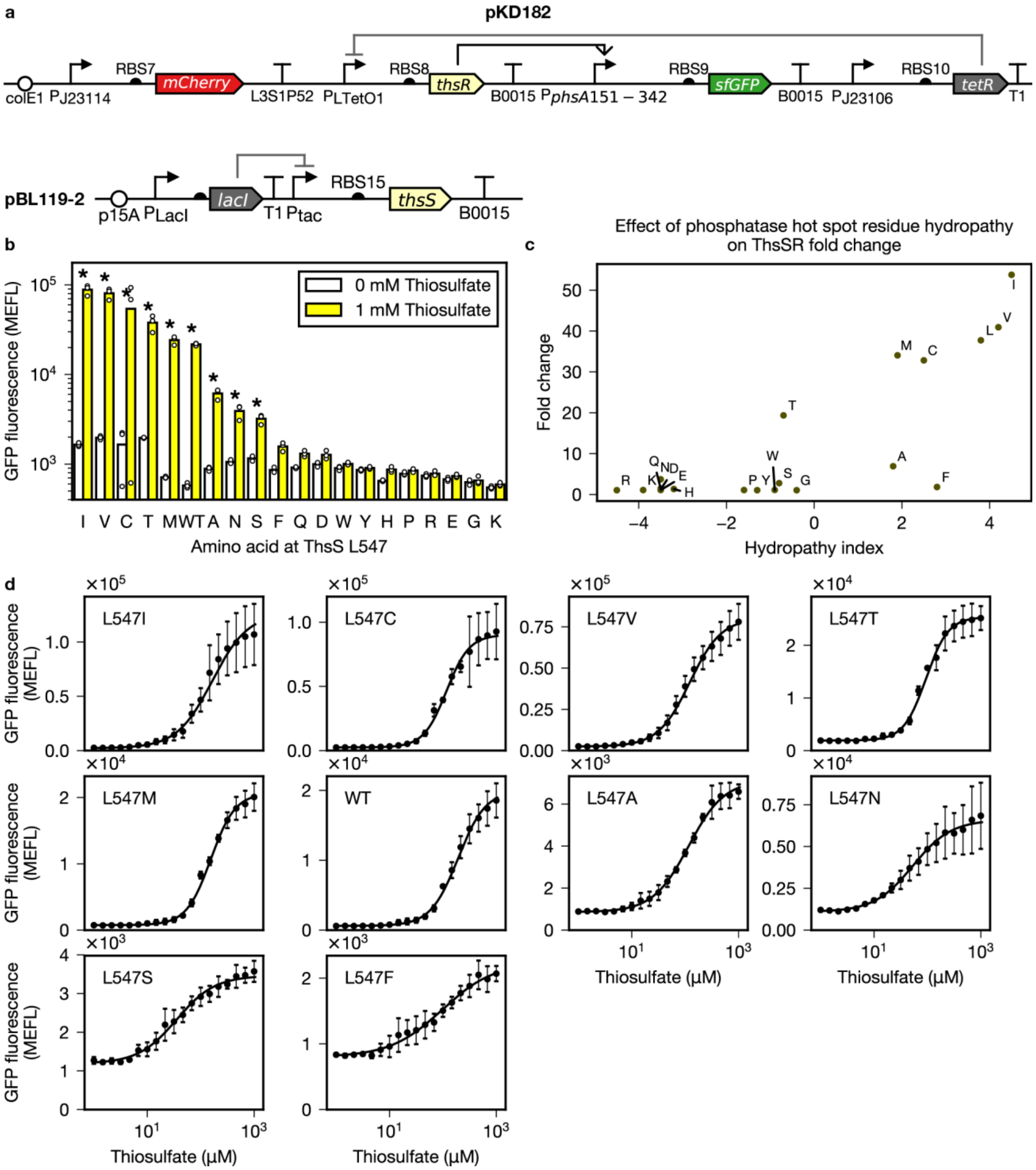

Supplementary Figure 11. Detailed characterization of ThsS phosphatase hot spot mutants. (**a-d**) As described in **Supplementary Fig. 10** but for the thiosulfate-activated ThsSR system.

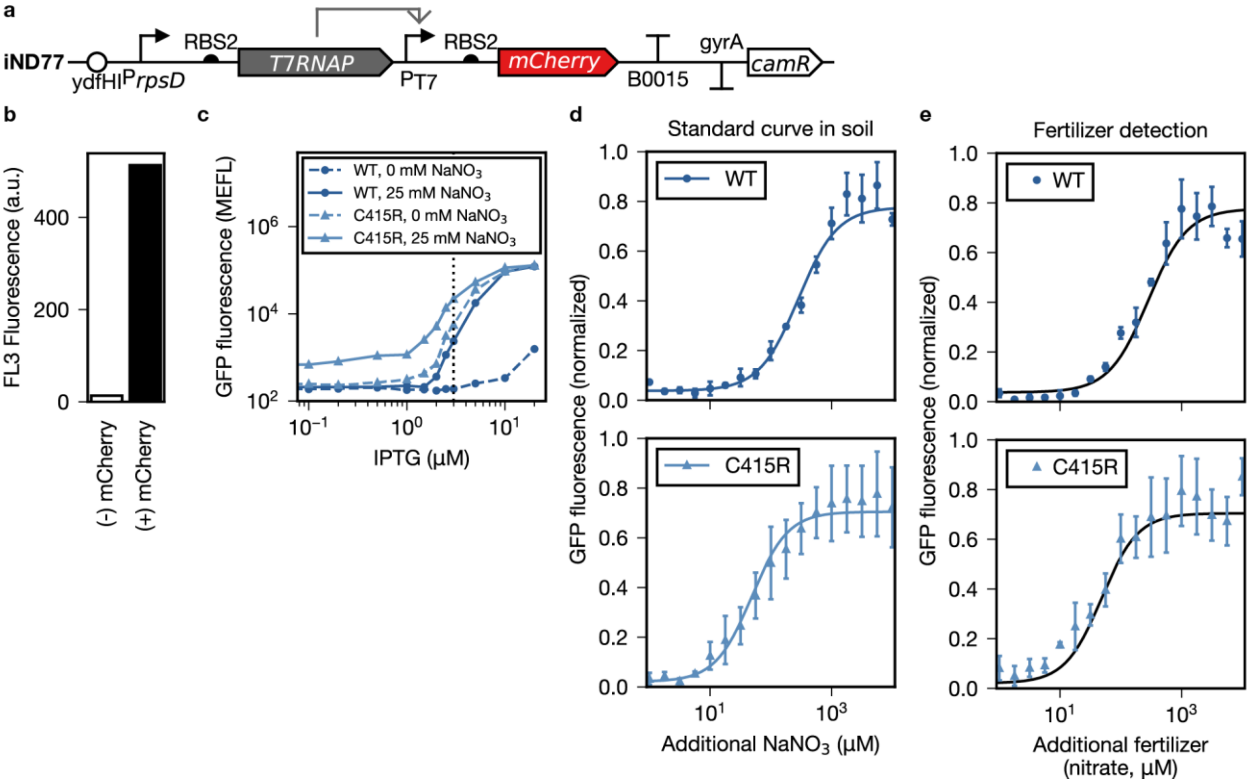

Supplementary Figure 12. Nitrate measurements in soil.

(**a**) Device schematic of iND77, which both knocks out YdfHI expression and overexpresses mCherry using T7 polymerase. To engineer the high and low fertilizer concentration soil sensor strains, iND77 was combined with iND138 (NarL-YdfI expression induction and sfGFP output) and iND27 (NarX expression) or iND71 (NarX(C415R) expression) (**Supplementary Fig. 3**). (**b**) Measurement of red fluorescence of *B. subtilis* with and without mCherry. (**c**) Expression level optimization of NarL-YdfI in the NarX and NarX(C415R) strains in soil (**Methods**). 0.3% xylose is used to induce NarX (dark blue circles) and NarX (C415R) (light blue circles). The dashed line indicates the conditions used for soil sensing experiments. (**d**) $NaNO_3$ transfer functions in soil of the NarX (top) and NarX(C415R) (bottom) TCSs. (**e**) Fertilizer transfer functions in soil of the NarX (top) and NarX(C415R) (bottom) TCSs with the transfer function fit from panel ‘d’. (**d, e**) Data points are the mean and error bars are as described in **Supplementary Fig. 6**. See **Supplementary Note 2** for additional details.

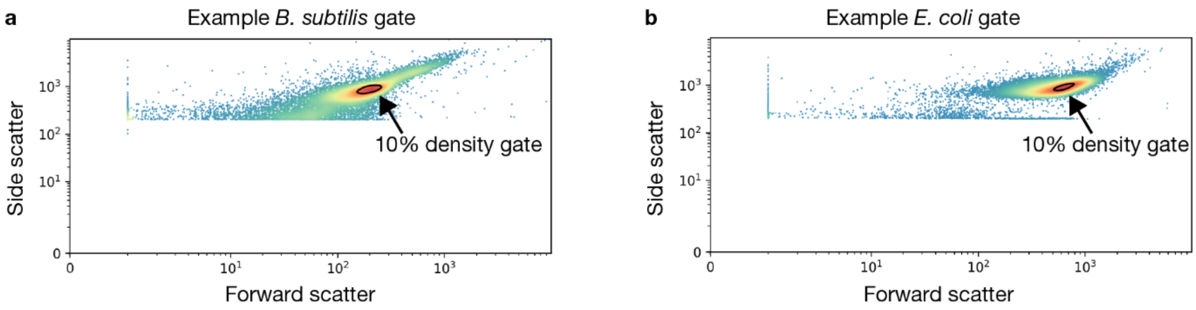

Supplementary Figure 13. Example of the flow cytometry gating strategy.

Representative scatter plots that show the result of the density gating algorithm used to analyze flow cytometry data (**Methods**) for both *B. subtilis* cells (**a**) and *E. coli* cells (**b**).

Supplementary References

1. Batchelor, E. & Goulian, M. Robustness and the cycle of phosphorylation and dephosphorylation in a two-component regulatory system. *Proc. Natl. Acad. Sci.* **100,** 691–696 (2003).
2. Gao, R. & Stock, A. M. Probing kinase and phosphatase activities of two-component systems in vivo with concentration-dependent phosphorylation profiling. *Proc. Natl. Acad. Sci.* **110,** 672–677 (2013).
3. Igoshin, O. A., Alves, R. & Savageau, M. A. Hysteretic and graded responses in bacterial two-component signal transduction. *Mol. Microbiol.* **68,** 1196–1215 (2008).
4. Serizawa, M. & Sekiguchi, J. The Bacillus subtilis YdfHI two-component system regulates the transcription of ydfJ, a member of the RND superfamily. *Microbiology* **151,** 1769–1778 (2005).
5. Zhu, Y. & Inouye, M. The role of the G2 box, a conserved motif in the histidine kinase superfamily, in modulating the function of EnvZ. *Mol. Microbiol.* **45,** 653–663 (2002).
6. Wolanin, P. M., Thomason, P. A. & Stock, J. B. Histidine protein kinases: key signal transducers outside the animal kingdom. *Genome Biol.* **3,** REVIEWS3013 (2002).
7. Kyte, J. & Doolittle, R. F. A simple method for displaying the hydropathic character of a protein. *J. Mol. Biol.* **157,** 105–132 (1982).
8. Daeffler, K. N.-M. *et al.* Engineering bacterial thiosulfate and tetrathionate sensors for detecting gut inflammation. *Mol. Syst. Biol.* **13,** 923 (2017).
9. Zhang, X.-Z. & Zhang, Y.-H. P. Simple, fast and high-efficiency transformation system for directed evolution of cellulase in Bacillus subtilis. *Microb. Biotechnol.* **4,** 98–105 (2011).

10. Trauth, S. & Bischofs, I. B. Ectopic Integration Vectors for Generating Fluorescent Promoter Fusions in Bacillus subtilis with Minimal Dark Noise. *PLOS ONE* **9,** e98360 (2014).
11. Britton, R. A. *et al.* Genome-Wide Analysis of the Stationary-Phase Sigma Factor (Sigma-H) Regulon of Bacillus subtilis. *J. Bacteriol.* **184,** 4881–4890 (2002).
12. Studier, F. W. & Moffatt, B. A. Use of bacteriophage T7 RNA polymerase to direct selective high-level expression of cloned genes. *J. Mol. Biol.* **189,** 113–130 (1986).
13. Chen, Y.-J. *et al.* Characterization of 582 natural and synthetic terminators and quantification of their design constraints. *Nat. Methods* **10,** 659–664 (2013).
14. Devi, S. N., Vishnoi, M., Kiehler, B., Haggett, L. & Fujita, M. In vivo functional characterization of the transmembrane histidine kinase KinC in Bacillus subtilis. *Microbiol. Read. Engl.* **161,** 1092–1104 (2015).
15. Espah Borujeni, A., Channarasappa, A. S. & Salis, H. M. Translation rate is controlled by coupled trade-offs between site accessibility, selective RNA unfolding and sliding at upstream standby sites. *Nucleic Acids Res.* **42,** 2646–2659 (2014).
16. Schmidl, S. R., Sheth, R. U., Wu, A. & Tabor, J. J. Refactoring and Optimization of Light-Switchable Escherichia coli Two-Component Systems. *ACS Synth. Biol.* **3,** 820–831 (2014).
17. Ramakrishnan, P. & Tabor, J. J. Repurposing Synechocystis PCC6803 UirS–UirR as a UV-Violet/Green Photoreversible Transcriptional Regulatory Tool in E. coli. *ACS Synth. Biol.* **5,** 733–740 (2016).
