# Supplementary material for: Phosphatase activity tunes two-component system sensor detection threshold
Source: Nat Commun. 2018 Apr 12;9:1433. doi: 10.1038/s41467-018-03929-y (PMC5897336; doi:10.1038/s41467-018-03929-y)
Supplement: Supplementary file 8 — Supplementary Software [file 41467_2018_3929_MOESM8_ESM.html]

Model


# Import Packages¶

In [1]:

```
%matplotlib inline
import numpy as np
import matplotlib.pyplot as plt
import matplotlib
import matplotlib.colors as colors
import pandas as pd
import lmfit
plt.style.use('landry-paper')

import ast
with open('colors.txt','r') as inf:
    color_scheme = ast.literal_eval(inf.read())
```

# Save Location¶

In [2]:

```
description = 'Model'
image_path = 'Plots/' + description+"/"
```

# Model Parameters¶

In [3]:

```
params = {}

# From Gao and Stock 2013
params['Cp'] = 4
params['Ct'] = 0.8
params['RRt'] = 10

# Generic Activating Hill function of form y = a + b * x^n/(Km^n + x^n)
params['a'] = 0.1
params['b'] = 0.9
params['n'] = 2
params['Km'] = 1

# Defaults of scalars to simulate mutations or input
params['phosphatase_scalar'] = 1
params['kinase_scalar'] = 1
params['kk_scalar'] = 1
params['kminusk_scalar'] = 1
```

# Model¶

In [4]:

```
# Calculates a transcription rate based upon model parameters and inducer concentration
def model(params):
    out = params.copy()
    
    # Simulate input by scaling either the autokinase or autophosphatase rates of the SK
    kk_scalar = params['kk_scalar']
    kminusk_scalar = params['kminusk_scalar']
    
    # Scale the bulk Cp parameter by auto kinase scalar and by the phosphatase scalar
    Cp = params['Cp'] * kk_scalar / params['phosphatase_scalar']
    Ct = params['Ct'] * kminusk_scalar / params['kinase_scalar']
    RRt = params['RRt']
    
    # Calculate the concentration of phosphorylated response regulator
    RRp = 0.5 * (Ct + Cp + RRt) - 0.5 * ((Ct + Cp + RRt)**2 - 4 * Cp * RRt)**0.5
    out['RRp'] = RRp
    
    # Calculate promoter activity
    a = params['a']
    b = params['b']
    n = params['n']
    Km = params['Km']
    Pa = a + b * RRp**n / (Km**n + RRp**n)
    out['Pa'] = Pa
    
    return out
```

# Compute Responses¶

In [5]:

```
# Do not vary SK kinase activity or SK autophosphatase activity
params['kinase_scalar'] = 1
params['kminusk_scalar'] = 1

# Simulate inducer induction by changing autokinase activity of the SK concentrations
xlim = (10**-2, 10**1)
params['kk_scalar'] = np.logspace(np.log10(xlim[0]), np.log10(xlim[-1]),100)
data_phosphatase = pd.DataFrame(index = pd.Index(params['kk_scalar'], name = "Input"))

# Plot transfer functions for different phosphatase activities
phosphatase_scalars = np.logspace(-2,2,25)
for phosphatase_scalar in phosphatase_scalars:
    params['phosphatase_scalar'] = phosphatase_scalar
    out = model(params)
    data_phosphatase[phosphatase_scalar*100] = out['Pa']
```

In [6]:

```
def compute_statistics(s):
    low = s.iloc[0]
    high = s.iloc[-1]
    dynamic_range = high/low
    half_maximum = low + (high-low)/2
    k = s.index[s>half_maximum].min()
    return pd.Series({'Dynamic Range':dynamic_range,'K':k})

analysis = data_phosphatase.apply(func = compute_statistics, axis = 0)
```

# Colors¶

In [7]:

```
# Plot colorbar
num = 101
phosphatase_scalars = [.01,.1,1]
color_lim = ['#bbbbbb','#000000']
cmap = matplotlib.colors.LinearSegmentedColormap.from_list('phosphatase', [color_lim[0],color_lim[-1]], N=num)
norm = matplotlib.colors.LogNorm(vmin=data_phosphatase.columns.min(), vmax=data_phosphatase.columns.max())
get_color = lambda pa: cmap(norm(pa))
```

# Plot Transfer Functions¶

In [8]:

```
# Create axes
fig = plt.figure(figsize = (3,3))
gs = matplotlib.gridspec.GridSpec(1,2)
ax = fig.add_subplot(gs[0,0])
ax_cbar = fig.add_subplot(gs[0,1])

# Plot colorbar
cb = matplotlib.colorbar.ColorbarBase(ax_cbar, cmap=cmap, orientation='vertical',
                                       label = "Phosphatase activity (% WT)",
                                       norm=norm)
ax_cbar.yaxis.set_label_position('right')


# Plot transfer functions for different phosphatase activities
for col in data_phosphatase[data_phosphatase.columns[range(0,25,3)]]:
    ax.plot(data_phosphatase.index, data_phosphatase[col], color = get_color(col), label = str(col)+'*C$_p$')

# Format Plot
ax.set_xscale('log')
ax.set_xlim(xlim)
ax.set_ylim((0,1));
ax.set_xlabel('Input')
ax.set_ylabel('Transcription', labelpad = 0)

height = 1.2
width = 1.2
cbar_width = 1
bbox_inches =  ax.get_position().transformed(fig.transFigure).transformed(fig.dpi_scale_trans.inverted())
bbox =  ax.get_position()
ax.set_position([bbox.x0,bbox.y0,bbox.width*width/bbox_inches.width,bbox.height*height/bbox_inches.height])
bbox2 = ax.get_position()
ax_cbar.set_position([bbox2.x1*1.02, bbox2.y0, 0.03, bbox2.height])

# Save Plots
fig.savefig(image_path + description + ' Transfer Functions.svg', bbox_inches = 'tight', transparent = True)
fig.savefig(image_path + description + ' Transfer Functions.png', bbox_inches = 'tight', transparent = False)
```

# Plot K1/2 Dynamic Range Relationship¶

In [9]:

```
# Create axes
fig = plt.figure(figsize = (3,3))
gs = matplotlib.gridspec.GridSpec(1,2)
ax = fig.add_subplot(gs[0,0])
ax_cbar = fig.add_subplot(gs[0,1])
cb = matplotlib.colorbar.ColorbarBase(ax_cbar, cmap=cmap, orientation='vertical',
                                       label = "Phosphatase activity (% WT)",
                                       norm=norm)
ax_cbar.yaxis.set_label_position('right')

for col in analysis:
    ax.plot(analysis.loc['K', col], analysis.loc['Dynamic Range', col], marker = 'o', color = get_color(col))
    
    
ax.set_xscale('log')
ax.set_xlim(xlim)
ax.set_ylim(ymin=0)
ax.set_xlabel('$K_{1/2}$ (Input)')
ax.set_ylabel('Dynamic Range', labelpad = 0)

height = 1.2
width = 1.2
cbar_width = 1
bbox_inches =  ax.get_position().transformed(fig.transFigure).transformed(fig.dpi_scale_trans.inverted())
bbox =  ax.get_position()
ax.set_position([bbox.x0,bbox.y0,bbox.width*width/bbox_inches.width,bbox.height*height/bbox_inches.height])
bbox2 = ax.get_position()
ax_cbar.set_position([bbox2.x1*1.02, bbox2.y0, 0.03, bbox2.height])

fig.savefig(image_path + "K, dynamic range Relationship.png", dpi = 300, bbox_inches = 'tight')
fig.savefig(image_path + "K, dynamic range Relationship.svg", transparent = True, bbox_inches = 'tight')
```

# Vary Kinase Activity¶

In [10]:

```
# Do not vary SK kinase activity or SK autophosphatase activity
params['phosphatase_scalar'] = 1
params['kk_scalar'] = 1

# Simulate inducer induction by changing autokinase activity of the SK concentrations
xlim = (10**-3, 10**0)
params['inducer'] = np.logspace(np.log10(xlim[0]), np.log10(xlim[-1]),100)
params['kminusk_scalar'] = params['inducer']**-1
data_kinase = pd.DataFrame(index = pd.Index(params['inducer'], name = "Input"))

# Plot transfer functions for different phosphatase activities
kinase_scalars = np.logspace(-2,2,25)
for kinase_scalar in kinase_scalars:
    params['kinase_scalar'] = kinase_scalar
    out = model(params)
    data_kinase[kinase_scalar*100] = out['Pa']
    
analysis_kinase = data_kinase.apply(func = compute_statistics, axis = 0)
```

In [11]:

```
# Create axes
fig = plt.figure(figsize = (3,3))
gs = matplotlib.gridspec.GridSpec(1,2)
ax = fig.add_subplot(gs[0,0])
ax_cbar = fig.add_subplot(gs[0,1])

# Plot colorbar
cb = matplotlib.colorbar.ColorbarBase(ax_cbar, cmap=cmap, orientation='vertical',
                                       label = "Kinase activity (% WT)",
                                       norm=norm)
ax_cbar.yaxis.set_label_position('right')


# Plot transfer functions for different phosphatase activities
for col in data_kinase[data_kinase.columns[range(0,25,3)]]:
    ax.plot(data_kinase.index, data_kinase[col], color = get_color(col), label = str(col)+'*C$_p$')

# Format Plot
ax.set_xscale('log')
ax.set_xlim(xlim)
ax.set_ylim((0,1));
ax.set_xlabel('Input')
ax.set_ylabel('Transcription', labelpad = 0)

height = 1.2
width = 1.2
cbar_width = 1
bbox_inches =  ax.get_position().transformed(fig.transFigure).transformed(fig.dpi_scale_trans.inverted())
bbox =  ax.get_position()
ax.set_position([bbox.x0,bbox.y0,bbox.width*width/bbox_inches.width,bbox.height*height/bbox_inches.height])
bbox2 = ax.get_position()
ax_cbar.set_position([bbox2.x1*1.02, bbox2.y0, 0.03, bbox2.height])

# Save Plots
fig.savefig(image_path + description + ' Kinase Transfer Functions.svg', bbox_inches = 'tight', transparent = True)
fig.savefig(image_path + description + ' Kinase Transfer Functions.png', bbox_inches = 'tight', transparent = False)
```

In [12]:

```
# Create axes
fig = plt.figure(figsize = (3,3))
gs = matplotlib.gridspec.GridSpec(1,2)
ax = fig.add_subplot(gs[0,0])
ax_cbar = fig.add_subplot(gs[0,1])
cb = matplotlib.colorbar.ColorbarBase(ax_cbar, cmap=cmap, orientation='vertical',
                                       label = "Kinase activity (% WT)",
                                       norm=norm)
ax_cbar.yaxis.set_label_position('right')

for col in analysis_kinase:
    ax.plot(analysis_kinase.loc['K', col], analysis_kinase.loc['Dynamic Range', col], marker = 'o', color = get_color(col))
    
    
ax.set_xscale('log')
ax.set_xlim(xlim)
ax.set_ylim(ymin=0)
ax.set_xlabel('$K_{1/2}$ (Input)')
ax.set_ylabel('Dynamic Range', labelpad = 0)

height = 1.2
width = 1.2
cbar_width = 1
bbox_inches =  ax.get_position().transformed(fig.transFigure).transformed(fig.dpi_scale_trans.inverted())
bbox =  ax.get_position()
ax.set_position([bbox.x0,bbox.y0,bbox.width*width/bbox_inches.width,bbox.height*height/bbox_inches.height])
bbox2 = ax.get_position()
ax_cbar.set_position([bbox2.x1*1.02, bbox2.y0, 0.03, bbox2.height])

fig.savefig(image_path + "Kinase K, dynamic range Relationship.png", dpi = 300, bbox_inches = 'tight')
fig.savefig(image_path + "Kinase K, dynamic range Relationship.svg", transparent = True, bbox_inches = 'tight')
```

# Save Figure Data¶

In [13]:

```
# Create Folder

figure = 'S1'

import os
figure_data_dir = 'Figure Data/Figure ' + figure
if not os.path.exists(figure_data_dir):
    os.makedirs(figure_data_dir)
```

In [14]:

```
# Save Figure Datapoints
writer = pd.ExcelWriter(figure_data_dir + '\Figure Data.xlsx', engine='xlsxwriter')

formatted_analysis = analysis.transpose()
formatted_analysis.index.name = 'Phosphatase Activity (% WT)'
formatted_analysis.to_excel(writer, sheet_name='Phosphatase Data')


formatted_analysis = analysis_kinase.transpose()
formatted_analysis.index.name = 'Kinase Activity (% WT)'
formatted_analysis.to_excel(writer, sheet_name='Kinase Data')

writer.save()
```

In [ ]:

```

```
